# IR-MALDI Mass Spectrometry Imaging with Plasma Post-Ionization of Nonpolar Metabolites

**DOI:** 10.1021/acs.analchem.2c03247

**Published:** 2022-11-10

**Authors:** Julian Schneemann, Karl-Christian Schäfer, Bernhard Spengler, Sven Heiles

**Affiliations:** † Institute of Inorganic and Analytical Chemistry, Justus Liebig University Giessen, 35392 Giessen, Germany; ‡ TransMIT GmbH, 35392 Giessen, Germany; § Leibniz-Institut für Analytische Wissenschaften - ISAS - e.V., Otto-Hahn-Straße 6b, 44139 Dortmund, Germany; ∥ Lipidomics, Faculty of Chemistry, University of Duisburg-Essen, Universitätsstrasse 5, 45141 Essen, Germany

## Abstract

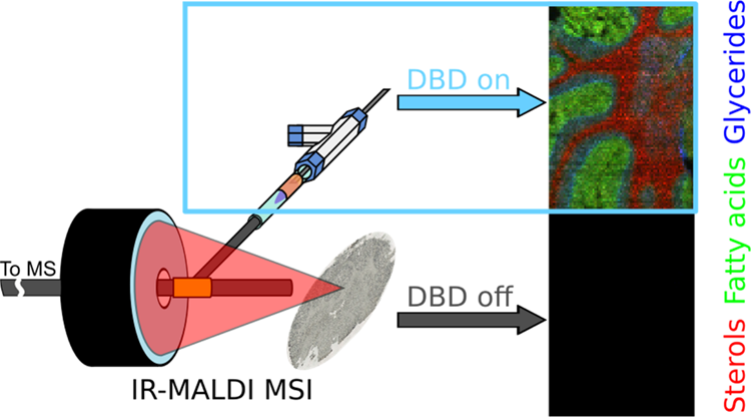

Ambient mass spectrometry imaging (MSI) methods come with the advantage of visualizing biomolecules from tissues with no or minimal sample preparation and operation under atmospheric-pressure conditions. Similar to all other MSI methodologies, however, ambient MSI modalities suffer from a pronounced bias toward either polar or nonpolar analytes due to the underlying desorption and ionization mechanisms of the ion source. In this study, we present the design, construction, testing, and application of an in-capillary dielectric barrier discharge (DBD) module for post-ionization of neutrals desorbed by an ambient infrared matrix-assisted laser desorption/ionization (IR-MALDI) MSI source. We demonstrate that the DBD device enhances signal intensities of nonpolar compounds by up to 10^4^ compared to IR-MALDI without affecting transmission of IR-MALDI ions. This allows performing MSI experiments of mouse tissue and *Danaus plexippus* caterpillar tissue sections, visualizing the distribution of sterols, fatty acids, monoglycerides, and diglycerides that are not detected in IR-MALDI MSI experiments. The pronounced signal enhancement due to IR-MALDI-DBD compared to IR-MALDI MSI enables mapping of nonpolar analytes with pixel resolutions down to 20 μm in mouse brain tissue and to discern the spatial distribution of sterol lipids characteristic for histological regions of *D. plexippus*.

## Introduction

Revealing the distribution of biomolecules within the context of cells or tissues is a key target in bioanalytic research. This is because characteristic biomolecule distribution patterns allow distinguishing tissue types, tissue regions, and cells or monitoring disease progression.^1^ In addition to these phenomenological approaches for tissue diagnostics, which mostly rely on databases and computer-guided decision making, unraveling biomolecule distributions also holds the promise to rationalize underlying mechanisms of biomolecule organization and spatially confined biochemical events.^2−4^

For this reason, recent years have seen a rapid development of bioanalytical platforms that are capable to locally interrogate bimolecular abundances. One of these tools is mass spectrometry imaging (MSI).^5^ All MSI technologies combine the sensitivity, mass resolution, and mass accuracy of modern mass spectrometers with ion sources that are capable to locally desorb and ionize biomolecules. Well-established methods are matrix-assisted laser desorption/ionization (MALDI) with UV laser light or secondary ion mass spectrometry (SIMS) that provide lateral resolutions down to the single-cell level, with no or moderate biomolecule fragmentation, respectively.^6^ However, these performance characteristics come with the cost of matrix application in UV-MALDI and fixation as well as transfer into the vacuum in SIMS. Therefore, ambient MSI methods have emerged that require no or minimal sample preparation.^7^ This includes methods such as desorption electrospray ionization (DESI),^8^ nanoDESI,^9^ dielectric barrier discharge (DBD)/low-temperature plasma (LTP),^10^ or IR-MALDI.^11−14^ Even though most MSI methodologies can interrogate the distributions of multiple biomolecule classes, the underlying desorption and ionization mechanisms dictate desorption/ionization efficiencies. These fundamental mechanisms will ultimately determine the overall number of ions generated from the sample surface. Consequently, the lateral resolution and scope of ionized compounds are limited by the ion source choice. For example, Boskamp and Soltwisch recently showed that MALDI MSI of artificial phospholipid mixtures yields depleted phosphatidylethanolamine (PE) signals in the presence of phosphatidylcholines (PCs) with 0.31 wt % concentration or higher, i.e., an indication for ion suppression.^15^ Similar ion suppression effects or diminished ion yields for selected compound classes are also observed for other MSI modalities such as DESI^16^ or SIMS.^17^

For this reason, numerous groups have coupled primary ion sources with post-ionization setups. The goal is to influence the primary ionization mechanism or combine ion sources with orthogonal means of ionization to maximize the number of detected analytes. For example, Dreisewerd, Soltwisch, and co-workers established MALDI-2. This method relies on a second laser that irradiates the ablation plume of the primary MALDI laser to enhance analyte signals, number of detected compound classes, and increase protonated/deprotonated signals compared to all other molecular ions.^18^ Another method for laser-based post-ionization is resonance-enhanced multiphoton ionization (REMPI). As REMPI post-ionization results depend on the electronic structure of the analyte, this method has been used to increase ion yields of selected compounds after SIMS^19^ or UV-MALDI.^20^

Ambient MSI sources have also been combined with post-ionization methods. This includes vis/UV laser desorption/ionization (LDI). LDI allows for matrix-free sample preparation, but ion yields are lower than those in MALDI. To counteract the low intrinsic ion yield of LDI, numerous post-ionization schemes have been adopted.^21^ For example, Shiea et al. and later Murray and co-workers combined desorption/ionization by UV laser light with electrospray post-ionization of an ablated sample material.^53,54^ Yin et al. have demonstrated the use of UV laser ablation and vacuum UV photoionization for visualizing exogenous compounds in single cells.^22^ The material ejected upon vis/UV laser irradiation of tissues can also be post-ionized with plasma-based ion sources. Numerous groups have developed corresponding ambient MSI sources. For example, Lu et al. have developed a 532 nm laser-ablation DBD MSI source capable to visualize metabolites in the medicinal plant *Rheum palmatum* and in zebrafish.^23^ Very similar setups have been reported by Moreno-Pedraza et al.^24^ and Fowble et al.^25^ using 405 nm with LTP and 213 nm with direct analysis in real time, respectively.

Another means for laser-based sampling of biological tissues is IR-MALDI. Unlike vis/UV-LDI setups that have few chromophores in tissues, water mainly serves as a chromophore in IR-MALDI. This also helps to boost ion yields for polar compounds compared to vis/UV-LDI and mostly prevents analyte fragmentation. This is documented by desorption/ionization of intact proteins as well as nucleic acids of up to 100 kDa in the first reported IR-MALDI MS studies.^13,26^ Even IR-MALDI MSI studies of metabolites with lateral resolutions down to 25 μm have been reported.^11^ However, the analytic sensitivity of IR-MALDI is well below the performance of UV-MALDI.^27^ For this reason, IR-MALDI has been extended with post-ionization devices. Arguably best known is the combination of IR-MALDI with ESI, called IR-MALDESI or LAESI.^28,29^ IR-MALDESI has been pioneered and developed by Muddiman and co-workers, now achieving lateral resolutions of down to 50 μm.^30^ Instead of ESI, IR-MALDI has recently been combined with atmospheric-pressure chemical ionization to study the fungicide metabolism in plants.^31^ Other groups have combined IR-MALDI with laser-based post-ionization methods. For example, Kostiainen and co-workers achieved sub-100 μm lateral resolution and coverage of a broad spectrum of metabolites using IR-MALDI in combination with atmospheric-pressure photoionization.^32^

In this manuscript, we report the extension of an IR-MALDI MSI source with a newly developed in-line capillary DBD setup for post-ionization to extend the set of metabolites detectable in one MSI experiment. First, the design, construction, and performance tests will be detailed, followed by investigations of authentic standards and well-characterized samples to evaluate the analytical performance of the new ion source. To demonstrate the capabilities of the developed MSI setup, compounds exhibiting increased signal intensities with an activated DBD unit compared to IR-MALDI MSI were imaged in the mouse brain and *Danaus plexippus* caterpillar sections with down to 20 μm lateral resolution.

## Materials and Methods

### Materials

Deoxycholic acid (≥98%, HPLC) was purchased from Sigma-Aldrich and used without purification. Ethanol (Uvasol, Merck, Darmstadt, Germany) and water (LC-MS grade, VWR International GmbH, Darmstadt, Germany) were of LC-MS grade or higher. Ergosterol (≥85%) was obtained from Cayman chemical company. Helium was purchased from Praxair with a purity of 99.9999%. Mouse brain tissue (C57BL6/N male and female mice, 12–20 weeks of age) was obtained from collaboration partners at JLU Giessen (Institute of Veterinary Anatomy, Histology and Embryology, JLU Giessen) and stored at −80 °C until sectioning. Larvae of *D. plexippus* were raised on *A. curassavica*, provided by the Institute for Insect Biotechnology (Justus Liebig University Giessen, Giessen, Germany), and stored at −80 °C until sectioning. Chili pepper “Carolina Reaper” seeds were purchased from Chili Food (Bad Dürkheim, Germany), grown, collected, and dried. Paracetamol was analyzed from a tablet (Paracetamol-ratiopharm 500 mg).

### Sample Preparation

Sectioning of the mouse brain was performed with a cryotome (HM525; Thermo Scientific, Dreieich, Germany) at −20 °C. The resulting tissue sections of 20 μm thickness were thaw-mounted on glass slides and stored at −80 °C until further use. Before experiments, tissues sectioned were allowed to equilibrate to room temperature in a desiccator. Optical images of the samples were recorded before and after MSI experiments with a digital optical microscope (VHX-5000 digital microscope, Keyence Deutschland GmbH, Neu-Isenburg, Germany). For experiments with deoxycholic acid, the authentic standard was dissolved in 8:2 ethanol/water with a mass concentration of 1.6 mg/μL and 150 μL of the resulting solution was deposited on a microscopy slide with 10 μL/min flow rate using a dedicated ultrafine pneumatic sprayer (“SMALDIPrep”, TransMIT GmbH, Giessen, Germany). To study the chili pepper, the dried pepper was opened with a scalpel and glued to a MALDI target with a double-sided tape, so that the opened face of the chili was facing the inlet capillary of the mass spectrometer.

### Mass Spectrometry Imaging and Post-Ionization

All experiments were performed with a previously described home-built atmospheric-pressure IR-MALDI MSI source.^11^ More details are included in the Supporting Information.

The IR-MALDI MSI setup was modified to allow for dielectric barrier discharge (DBD) post-ionization within the mass spectrometric inlet capillary.^33,34^ The DBD was operated with a 10 kV AC voltage at a frequency of 3.2 kHz provided via an ignition coil (Accel Super Stock 8140C (Cleveland)) and power supply (McPower LAB-2305), driven by an inlet pulse of a square waveform generator (Rigol DG1022 (Preetz, Germany)) providing variable ignition voltages of 0–15 V (labeled as U throughout the manuscript).^35^ The He flow rate of the discharge gas was varied between 0 and 40 L/h. The direct flow of He into the vacuum of the mass spectrometer did not compromise the quality of the vacuum up to a flow of 40 L/h He.

The modified IR-MALDI-DBD post-ionization source was coupled to an orbital trapping mass spectrometer (Q Exactive, Thermo Fisher Scientific GmbH, Bremen, Germany). Complete instrument calibration was performed before source installation, and [PC 34:1 + K]^+^ (*m*/*z* 798.5415) and/or a polysiloxane signal (e.g., [(C_2_H_6_SiO)_5_ + H]^+^; *m*/*z* 371.1012) were used for internal mass calibration. The Q Exactive Orbitrap was operated in positive-ion mode, and settings were as follows: acceleration voltage 3 kV, S-lens 70, mass resolution 140,000 at *m*/*z* 200, inlet time 500 ms, higher-energy collisional dissociation (HCD) normalized collision energy setting between 10 and 40, automatic gain control disabled, and capillary temperature 350 °C.

### Data Processing and Data Analysis

For manual analysis of individual or averaged mass spectra, the Thermo XCalibur Qual Browser was used. MS images were processed using Mirion imaging software.^36^ The intensity scale of Thermo Fisher Scientific mass spectrometers, the normalized level (NL), is used throughout the manuscript as an absolute intensity scale. As the inlet time was the same in all experiments, NL values can be compared between measurements. All images were created with a bin width of 0.01 u and a maximum absolute *m*/*z* variance of 0.01 u. Ion signal intensities were normalized to the total ion count (TIC) per pixel and the highest signal intensity per image. Most signal assignments were based on accurate mass measurements and comparison to the LIPID MAPS,^37^ Metlin,^38^ or HMDB^39^ databases with an absolute mass error of less than 5 ppm. Signals matching multiple database entries were annotated as those compounds with the smallest mass error. On-tissue IR-MALDI MS^2^ experiments were performed for selected signals, and their results are reported in the Supporting Information.

## Results and Discussion

### Ion Source Design and Parameter Optimization

**Figure 1:**
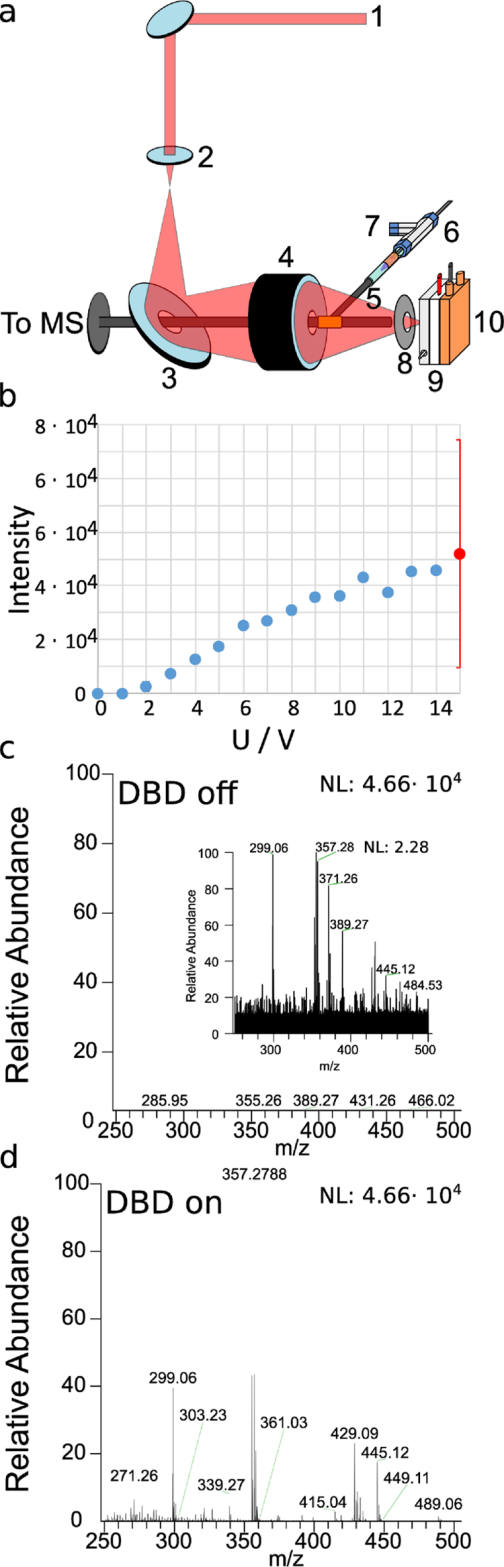
(a) Setup of the IR-MALDI MSI source with DBD post-ionization with (1): 2.94 μm laser beam, (2): prefocusing lens, (3): centrally bored mirror, (4): centrally bored objective lens, (5): transfer capillary with brass T-piece, (6): plasma torch, (7): helium supply, (8): aperture, (9): sample support with Peltier element, and (10): translation stage. Parameter optimization with homogenously sprayed deoxycholic acid [M–2H_2_O + H]^+^ for varying (b) the primary voltage U signal of [M–2H_2_O + H]^+^ upon IR-MALDI with (c) deactivated and (d) activated DBD module. The intensity scale maximum in (c) and (d) is 4.66 × 10^4^.

**Figure 2:**
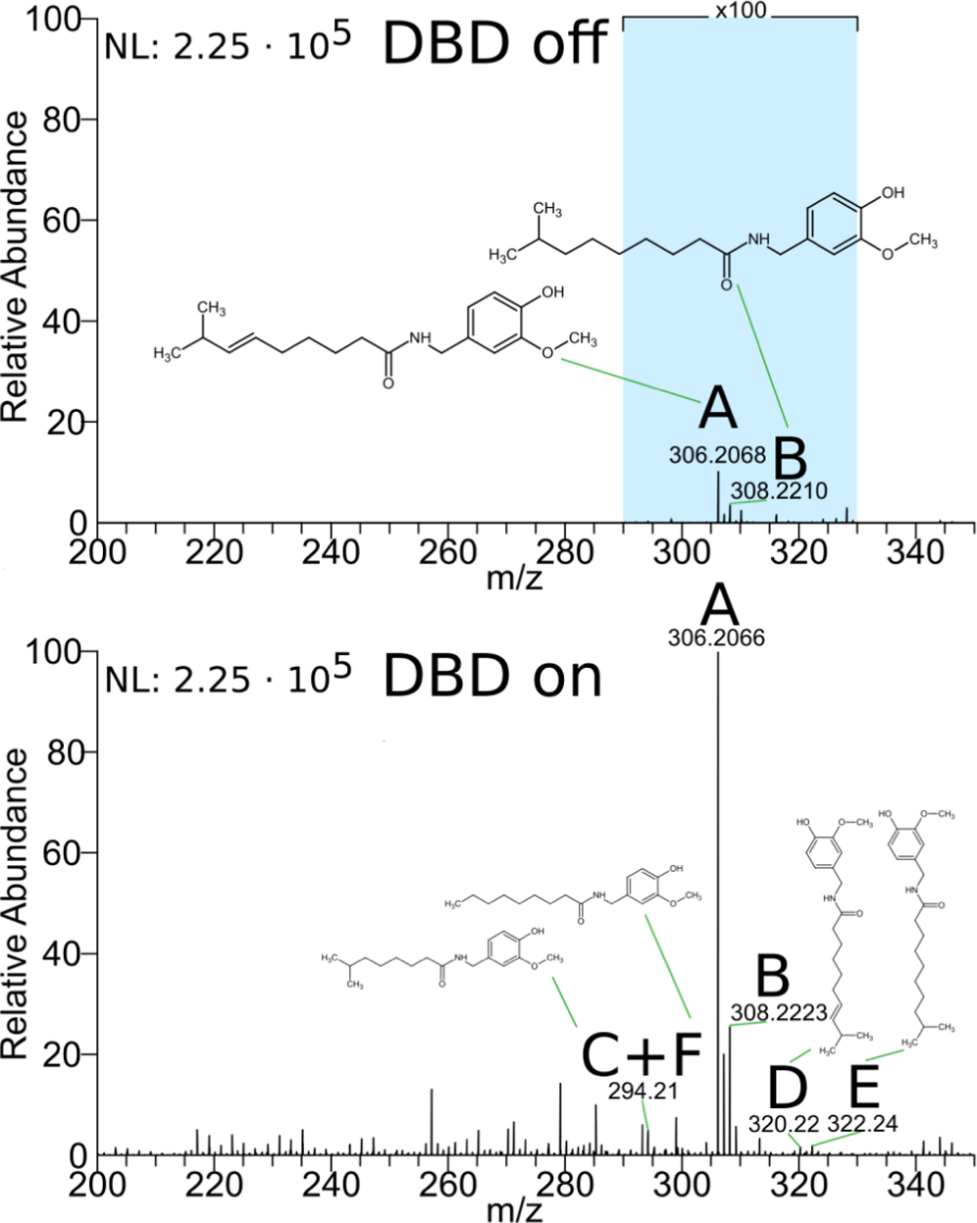
Sum of 150 mass spectra of chili pepper “*Carolina Reaper*” in laser-only mode (top) and laser + DBD mode (bottom) with (A) [capsaicin + H]^+^, (B) [dihydrocapsaicin + H]^+^, (C) [nordihydrocapsaicin + H]^+^, (D) [homocapsaicin + H]^+^, (E) [homodihydrocapsaicin + H]^+^, and (F) [nonivamide + H]^+^. The corresponding absolute NL values are 2.25 × 10^5^ for scale value 100.

**Figure 3:**
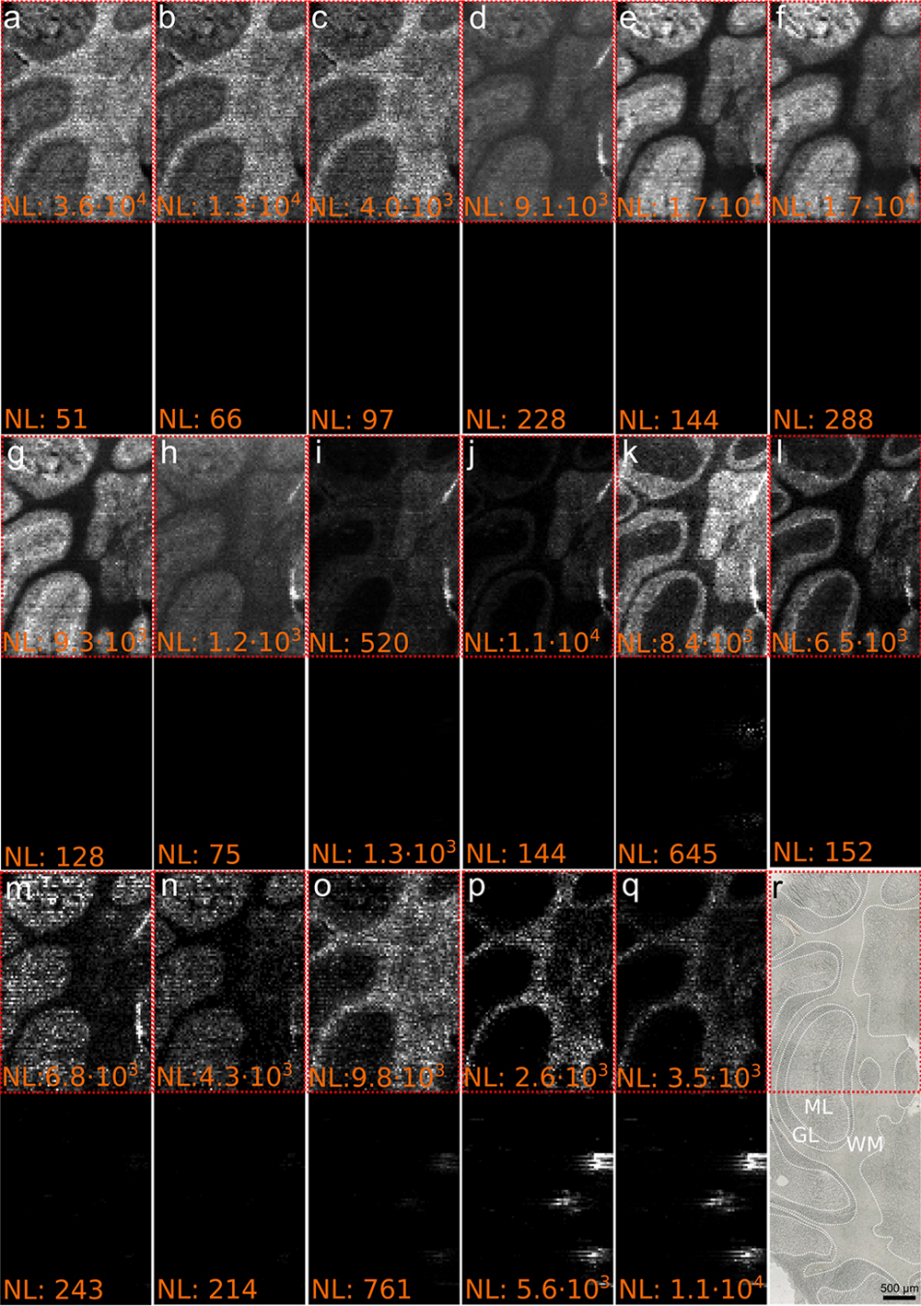
IR-MALDI-DBD (top rows) and IR-MALDI (bottom rows) MS images of the mouse brain, measured with 33 μm step size of (a) [cholesterol-H_2_O + H]^+^ (*m*/*z* 369.3516), (b) [cholesterol + H]^+^ (*m*/*z* 387.3621), (c) [cholecalciferol + H]^+^ or [desmosterol + H]^+^ (*m*/*z* 385.3465), (d) [palmitic acid + H]^+^ (*m*/*z* 257.2475), (e) [stearic acid + H]^+^ (*m*/*z* 285.2789), (f) [arachidonic acid + H]^+^ (*m*/*z* 305.2475), (g) [docosahexaenoic acid + H]^+^ (*m*/*z* 329.2475), (h) [docosatetraenoic acid + H]^+^ (*m*/*z* 333.2788), (i) [MG P-18:2 + H]^+^ (*m*/*z* 339.2893), (j) [MG 18:1 + H]^+^ (*m*/*z* 357.2999), (k) [MG 20:4 + H]^+^ (*m*/*z* 379.2842), (l) [MG 22:6 + H]^+^ (*m*/*z* 403.2842), (m) [DG P-32:1 + H]^+^ (*m*/*z* 551.5033), (n) [DG P-34:1 + H]^+^ (*m*/*z* 579.5346), (o) [DG P-36:2 + H]^+^ (*m*/*z* 605.5503), (p) [HexCer(d18:0/22:2) + H]^+^ (*m*/*z* 782.6504), (q) [HexCer(d18:1:24:1) + H]^+^ (*m*/*z* 810.6817), and (r) optical image prior MSI. GL: granular layer, ML: molecular layer, WM: white matter. The red dotted rectangles indicate the part of the tissue scanned with IR-MALDI-DBD MSI. The NL for the top and bottom rows is shown in orange.

**Figure 4:**
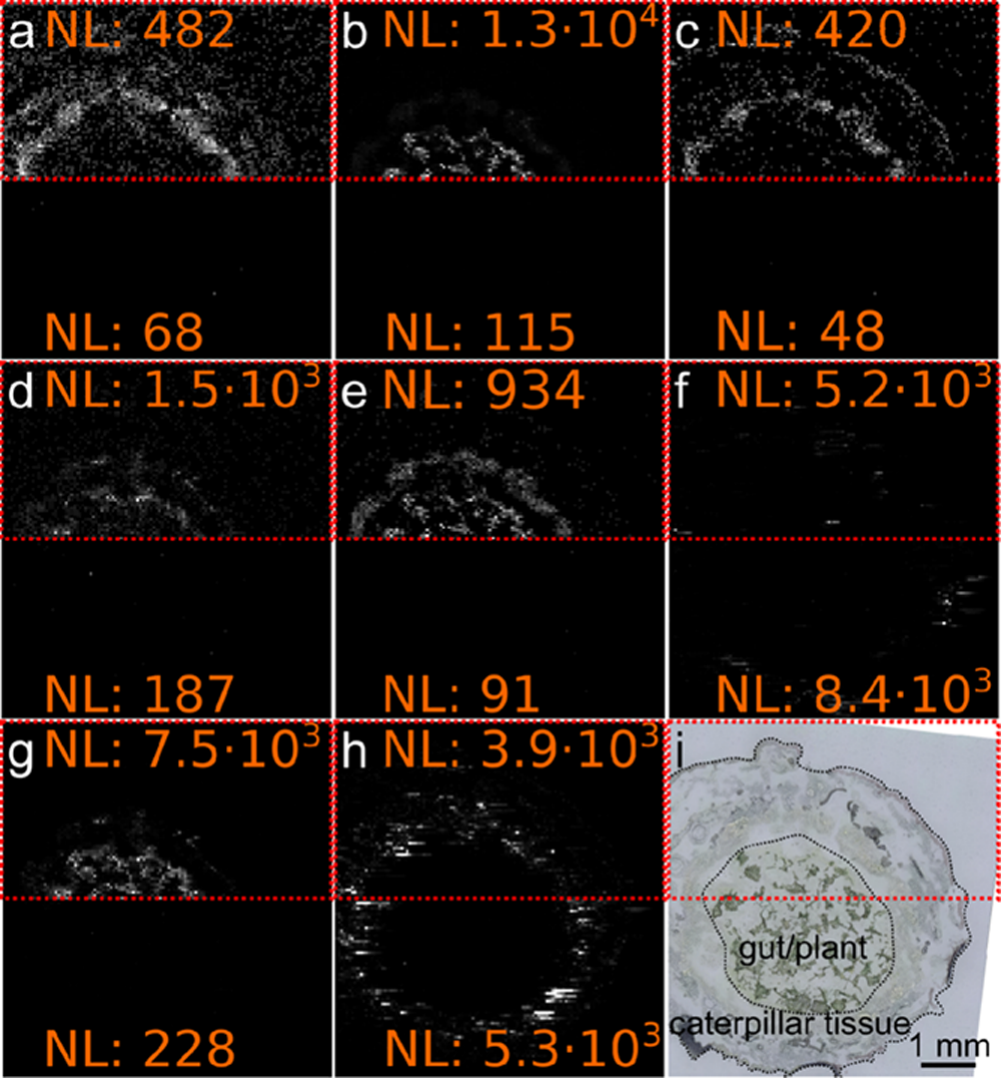
IR-MALDI-DBD (top rows) and IR-MALDI (bottom rows) MS images of a transversal cross section of monarch butterfly (*Danaus plexippus*) caterpillar, fed on tweedia (*Oxypetalum coeruleum*), with a step size of 50 μm. MS images (a–i). (a) [C_27_H_45_O]^+^ (*m*/*z* 385.3465), (b) [C_30_H_51_O]^+^ (*m*/*z* 427.3934), (c) [C_28_H_47_O]^+^ (*m*/*z* 399.3621), (d) [C_27_H_43_O]^+^ (*m*/*z* 383.3308), (e) [C_29_H_47_O]^+^ (*m*/*z* 411.3621), (f) [C_29_H_47_O_3_]^+^ (*m*/*z* 465.3339), (g) [C_30_H_49_O]^+^ (*m*/*z* 425.3778), (h) [C_42_H_80_NO_8_P + K]^+^ (*m*/*z* 796.5253), (i) optical image of the tissue section before MSI experiments. The red dotted rectangles indicate the part of the tissue scanned with IR-MALDI-DBD MSI. NL values for the top and bottom rows are shown in orange. Some of the signals were assigned: (a) vitamin D3, previtamin D3, cholest-4-en-3-one, desmosterol, and 7-dehydrocholesterol; (b) lanosterol, cycloartenol, cycloeucalenol, ß-amyrin, and lupeol; (g) lupenone. Potassiated PC(34:2) is shown in panel (h) for comparison.

With the goal to maximize the coverage of metabolite and lipid compound classes in a single ambient MSI experiment, an IR-MALDI MSI setup was combined with an in-source DBD ionization unit. The new IR-MALDI ion source, schematically shown in Figure 1a, is based on the setup previously reported by our group^11^ and was adapted for in-source DBD experiments. In short, IR-MALDI is performed with a Nd:YAG-pumped OPO system emitting light at 20 Hz repetition rate with a wavelength of 2.94 μm and a laser pulse width of 6 ns. The laser light (Figure 1a, 1) is guided via mirrors into a prefocusing lens (Figure 1a, 2), reflected by a mirror containing a central bore (Figure 1a, 3) for the MS inlet capillary, and focused with a dedicated IR-focusing objective lens with a numerical aperture of 0.4 (TransMIT GmbH, Giessen, Germany) (Figure 1a, 4) through an aperture (Figure 1a, 8) onto a Peltier-cooled sample, mounted on an xyz-movable stage (Figure 1a, 9,10). The MS inlet capillary is arranged in the center of the focusing objective to maximize the capture of desorbed neutrals and ions from the sampled surface.^40^ The MS inlet capillary is equipped with a T-piece, which connects the setup to the DBD ion source (Figure 1a, 5–7). The DBD plasma is operated with He gas, and reactive species are transported into the MS inlet capillary to interact with desorbed/ionized molecules. Importantly, the desorbed analytes do not directly interact with the DBD plasma but with the reactive species generated in the plasma. Molecules ionized during IR-MALDI or DBD are transported into an orbital trapping mass analyzer and are simultaneously detected.

To optimize the performance of the developed setup, deoxycholic acid was sprayed onto a sample holder and cooled to 14.5 °C, and the corresponding [M–2 H_2_O + H]^+^ signal intensity (*m*/*z* 357.2788) was monitored as a function of the primary DBD voltage, He flow rate, and DBD duty cycle. The duty cycle was defined as the time fraction during which the square waveform was active. The results of these experiments are shown in Figure 1 and S3a,b. Pronounced intensity fluctuations of the signal were observed due to the nonuniform crystallization of the analytic standard (Figure S1). Without the DBD device being activated, the signal of *m*/*z* 357.2788 in positive-ion mode was close to zero, indicating that deoxycholic acid is not efficiently ionized by IR-MALDI. Activation of the DBD module and varying the He flow rate between 0 and 40 L/h with U at 13 V and the duty cycle at 50% resulted in deoxycholic acid-associated mass spectrometric signals that increased to ∼6 × 10^4^ at 20 L/h He flow and started to decrease for further increased He flow rates (Figure S3a). This indicates that active species created in the He plasma reach the capillary inlet region and allow post-ionization of desorbed neutral analytes. For He flow rates above 20 L/h, the excess gas from the DBD module most likely reduces the hydrodynamic flow around the inlet capillary that is responsible for capturing desorbed neutral analytes. The [M–2 H_2_O + H]^+^ signal intensities as functions of the DBD voltage (at a He flow rate of 20 L/h, duty cycle 50%) and duty cycle (at a He flow rate 20 L/h, DBD voltage 13 V) are shown in Figures 1b and S3b, respectively. Although the monitored mass spectrometric signal increases from 0 to 10 V and saturates at around 5 × 10^4^ for the highest accessible DBD voltage of 15 V, the duty cycle marginally affects the signal intensity. The optimized settings were used for all of the following experiments. While the IR-MALDI signal of *m*/*z* 357.2788 was only 2.17 (Figure 1c), activation of the DBD module operated with the optimized parameters resulted in a 20,000-fold increase of the signal to 4.66 × 10^4^. This demonstrates that desorbed neutrals are not or only to a small degree ionized by IR-MALDI and can be post-ionized in the modified inlet capillary system, allowing for coaxial IR-MALDI with DBD post-ionization.

### Signal Enhancement and Ambient Sampling Using IR-MALDI with DBD

To investigate the overall performance characteristics as well as signal enhancements achievable with the developed DBD inlet capillary and to test which molecules are preferentially ionized by the DBD module, authentic standards and well-characterized tissue sections were studied with the new IR-MALDI-DBD-MS setup. Selected results are shown in Figures 2, S4–S7, and Table 1. An analyte known to be ionized with plasma-based methods is capsaicin and related metabolites in chili pepper. These analytes have been studied by LTP and were shown to only efficiently desorb and ionize from tissue surfaces if the LTP plasma temperature is raised to about 100 °C.^41,42^ For this reason, a sliced chili pepper “*Carolina Reaper*” sample was selected as a model to study the effect of combining IR-MALDI as a means for desorption and ionization with in-capillary DBD post-ionization for mass spectrometric signal enhancement. IR-MALDI results without DBD post-ionization are shown in Figure 2, top. The signals at *m*/*z* 306.2066 (label A) and *m*/*z* 308.2223 (label B) were assigned to protonated capsaicin and dihydrocapsaicin. The maximum signal intensity of protonated capsaicin is 2.27 × 10^2^, documenting the capability of IR-MALDI to desorb capsaicin from the sample surface and ionize a part of the desorbed analytes during IR-MALDI. Despite the detection of capsaicin and dihydrocapsaicin ions in IR-MALDI experiments without DBD, the signal intensities of the compounds increased by about a factor of 1000 when switching on the DBD unit, as shown in Figure 2, bottom. This indicates that a large fraction of analytes is not ionized during IR-MALDI but is transported into the inlet capillary where the neutrals are post-ionized via DBD. Due to the signal enhancement when combining IR-MALDI and DBD, HCD can be performed, confirming the signal assignment as protonated capsaicin and dihydrocapsaicin (Figure S4). In addition to the signal enhancement of analytes already detected with IR-MALDI, activation of the DBD module resulted in the enhancement of other analyte signals by up to 3 orders of magnitude and above the noise level. These signals are labeled as C, D, E, and F in Figure 2 and were assigned as protonated nordihydrocapsaicin, homocapsaicin, homodihydrocapsaicin and nonivamide, respectively, metabolites known to be present in chili pepper.^43^ This indicates that the IR-MALDI-DBD setup can increase the coverage of detectable metabolites by boosting signal intensities compared to IR-MALDI.

**Table 1:** Ions and Corresponding Signal Intensities (NL) upon IR-MALDI and IR-MALDI + DBD for Authentic Standards, Chili Pepper, and Mouse Brain Tissue

substance	ion	IR-MALDI only (NL)	IR-MALDI + DBD (NL)	DBD enhancement of signal intensity
capsaicin	[M + H]^+^	2.27 × 10^2^	2.25 × 10^5^	9.9 × 10^2^
dihydrocapsaicin	[M + H]^+^	7.76 × 10^1^	5.72 × 10^4^	7.4 × 10^2^
nordihydrocapsaicin/nonivamide	[M + H]^+^	7.40	1.12 × 10^4^	1.5 × 10^3^
homocapsaicin	[M + H]^+^	1.92	3.70 × 10^3^	1.9 × 10^3^
homodihydrocapsaicin	[M + H]^+^	1.79	4.21 × 10^3^	2.4 × 10^3^
paracetamol	[M + H]^+^	1.86 × 10^1^	1.12 × 10^5^	6.0 × 10^3^
cholesterol	[M – H_2_O + H]^+^	2.36 × 10^1^	7.82 × 10^5^	3.3 × 10^4^
cholesterol	[M + H]^+^	7.74 × 10^–2^	3.41 × 10^3^	4.4 × 10^4^
cholecalciferol	[M + H]^+^	1.34 × 10^–1^	3.98 × 10^3^	3.0 × 10^4^
stearic acid	[M + H]^+^	N.D.	1.72 × 10^4^	N.A.
palmitic acid	[M + H]^+^	N.D.	9.06 × 10^3^	N.A.
arachidic acid	[M + H]^+^	N.D.	1.69 × 10^4^	N.A.
docosatetraenoic acid	[M + H]^+^	4.90 × 10^–2^	9.06 × 10^3^	4.4 × 10^4^
ergosterol	[M – H_2_O + H]^+^	5.82 × 10^2^	7.62 × 10^3^	1.2 × 10^1^
MG P-18:2	[M + H]^+^	3.97 × 10^–1^	2.44 × 10^3^	6.2 × 10^3^
DG P-32:1	[M + H]^+^	3.23 × 10^–1^	4.32 × 10^2^	1.3 × 10^3^

Increased signal intensities were also achieved for authentic standards and nonpolar lipids in mouse brain tissue sections, as summarized in Table 1. For example, the signal of protonated paracetamol increased from 1.86 × 10^1^ to 1.12 × 10^5^ by activating the DBD unit. Signals of nonpolar sterols and fatty acids (FAs) increased by up to 10^4^-fold or increased above the noise level, respectively, when performing IR-MALDI-DBD from mouse tissue sections as compared to IR-MALDI (Table 1). The observed signal enhancements allowed direct confirmation of compound annotations via MS^2^ experiments (Figures S4 and S5). In contrast to improving the signal intensities by DBD of compounds that are not efficiently ionized via IR-MALDI, the developed IR-MALDI-DBD setup did not influence signal intensities or intensity distributions of polar lipids, which were already efficiently desorbed and ionized during IR-MALDI. This is most likely due to the fact that desorption and post-ionization are decoupled, i.e., analytes not ionized upon IR-MALDI are ionized within the inlet capillary, while already ionized compounds are not or only minimally influenced by the species generated in the DBD unit. This is in contrast to the reported MALDI-2 post-ionization scheme where primary ionization and post-ionization are intertwined.^18,44^ The preference to ionize polar compounds via IR-MALDI^45^ and nonpolar compounds via DBD^46^ is consistent with the reported ionization efficiencies of the ionization modalities but is in contrast to a recently reported UV-MALDI-DBD setup in which MALDI-2-type post-ionization seems to occur even though the post-ionization device and primary MALDI source are spatially separated.^47^

### Visualizing Nonpolar Lipids in Mouse Brain Tissue with IR-MALDI-DBD MSI

To demonstrate that the developed IR-MALDI-DBD ion source does also allow tracking distributions of compounds in tissue sections, MSI experiments with mouse brain tissue were performed. Results are shown in Figures 3, S6, and S7. Without DBD post-ionization, IR-MALDI mass spectra of the cerebellum region of mouse tissue were dominated by signals assigned to phospholipid compounds (Figure S6a). For example, *m*/*z* 798.5416 and *m*/*z* 826.5723 were assigned to [PC 34:1 + K]^+^ and [PC 36:1 + K]^+^, respectively. The overall NL of the phospholipid *m*/*z*-region was about 7 × 10^3^ and was only marginally affected by activation of the DBD device (Figure S6a). This indicates that DBD does not or only to a small degree affect phospholipid ionization and their relative intensities. No or minimal additional phospholipid ionization or fragmentation occurred. In contrast, some signals in the *m*/*z* range from 325 to 385 increased. Although the signals for [cholesterol–H_2_O + H]^+^ (*m*/*z* 369.3516) and [cholecalciferol + H]^+^/[desmosterol + H]^+^ (*m*/*z* 385.3465) were detected with a NL of 4 × 10^2^ and 1 × 10^0^ in IR-MALDI mass spectra, their intensities increased up to 133-fold to NL 2 × 10^4^ and 2 × 10^2^ via IR-MALDI-DBD, respectively. A signal increase of similar magnitude from mouse liver homogenate, spiked with ergosterol, was observed for endogenous [cholesterol–H_2_O + H]^+^ and for added [ergosterol–H_2_O + H]^+^ (*m*/*z* 379.3359), as shown in Figure S6b. The cholesterol-derived ion signal was enhanced by about 67-fold, and the signal of the spiked compound was enhanced by a factor of 12. Observed signal enhancements for nonpolar compounds is consistent with reported preferences of DBD to effectively ionize metabolites mainly containing hydrocarbons and a small number of heteroatoms.

To demonstrate the capability to visualize compounds efficiently ionized with DBD post-ionization, selected MS images in the mouse cerebellum are shown in Figure 3. The corresponding structures can be found in Figure S8. The lower half of the tissue sections was measured with IR-MALDI and the upper half with IR-MALDI-DBD using 33 μm pixel size, 200 × 70 pixels, and unchanged MS settings. As expected from experiments with authentic standards, MS images of [cholesterol-H_2_O + H]^+^ (*m*/*z* 369.3516) and [cholecalciferol + H]^+^/[desmosterol + H]^+^ (*m*/*z* 385.3465)^48^ significantly increased in intensity due to DBD activation as shown in Figure 3a,c, respectively. A signal assigned as [cholesterol + H]^+^ (*m*/*z* 387.3621) and colocalized with *m*/*z* 369.3516 yielded an MS image with an activated DBD unit only (Figure 3b). These MS images reveal the upregulation/accumulation of both compounds in the white matter (WM) region of the mouse cerebellum compared to the surrounding tissue regions (Figure 3r). This is in line with recent literature reports by Griffiths and co-workers, using on-tissue derivatization.^49^ DBD post-ionization generates protonated FA ions otherwise not detected in positive-ion mode and only accessible in low abundance in negative-ion mode with IR-MALDI.^44^ In total, five mass spectrometric signals were assigned to protonated FAs, as shown in Figure 3d–h. This is also consistent with reports of free FAs in the mouse brain.^50^ The signal at *m*/*z* 285.2788 is assigned as [stearic acid + H]^+^ (Figure 3e). While IR-MALDI MSI did not result in a signal above the noise level and thus yielded a blank MS image for this analyte, activation of the DBD device led to MSI results revealing regions with elevated and depleted stearic acid abundances. The WM of the mouse cerebellum was found to be depleted in stearic acid, in contrast to the granular layer (GL) and molecular layer (ML) in which increased signal intensity for the protonated FA was recorded relative to the WM (Figure 3e). The other four putative FAs showed similar spatial intensity maps with increased signal intensities in the GL compared to the ML and low or no intensity in the WM region (Figure 3d, f–h). Other classes of glycerolipids that lack a polar head group are monoglycerides (MGs) and diglycerides (DGs). Intensities for MGs and DGs are typically lower than phospholipid ion signals, and [M + Na]^+^ and [M + K]^+^ dominate IR-MALDI mass spectra. This is different in IR-MALDI-DBD MSI, as shown in Figure 3i–o assigned to MGs and DGs, respectively. Visualization of the compound distributions was successful only with IR-MALDI-DBD MSI with the exception of Figure 3k, in which circular regions in the IR-MALDI MS image appeared. These features most likely were artifacts from water condensation on the sample during the measurement, locally boosting IR-MALDI signal intensities but not revealing a real lateral distribution of the compound. The same regions were also enhanced in intensity in Figure 3o,p. Unlike for FAs, the association of MGs and DGs with histological features of the tissue differed between the compounds. Some MGs were found localized mainly in the GL (Figure 3j–l), whereas [MG P-18:2 + H]^+^ exhibited higher intensities in the WM and GL (Figure 3i). DGs, on the other hand, were elevated in intensity in the ML compared to the surrounding tissue (Figure 3m,n), except for [DG P-36:2 + H]^+^ that showed increased intensities in the WM (Figure 3o). While most analytes which were enhanced in intensity by DBD activation were below *m*/*z* 700, some were higher in mass. In particular, two hexosylceramides (HexCer) were found in the WM of the mouse brain only after DBD post-ionization (Figure 3p,q). The found distribution in the WM of [HexCer(d18:1:24:1) + H]^+^ is in line with reported visualization of the same compound by MALDI-2 in the rat brain.^32^ These findings demonstrate that the newly developed IR-MALDI-DBD source helps to boost ion signals of nonpolar metabolites via DBD post-ionization in MSI experiments, thereby extending the chemical space, which can locally be interrogated. Similar results were obtained in slight oversampling mode with a step size of 20 μm, as shown in Figure S7.

### Localizing Sterols in *Danaus plexippus* Tissue Sections

To demonstrate the analytical capabilities of the new ion source in ionizing and tracking the distribution of nonpolar compounds, a *Danaus plexippus* caterpillar transversal section of the gut region, containing food from tweedia plants (*Oxypetalum coeruleum*), a feeding plant acceptable for caterpillars, was studied, and results are shown in Figures 4, S9, and S10.

The lower half of the section was investigated with IR-MALDI MSI, whereas the upper half was scanned with an activated DBD unit. As sterols are a major class of lipids involved in the metabolism of all eucaryotic cells, most likely differing between the monarch butterfly caterpillar and the tweedia plant, mass spectrometric features in the *m*/*z* range 380–470 are shown in Figure 4. An optical image of the measured area can be found in Figure 4i. All of these ions, except for Figure 4f,h, were absent in MS images recorded with IR-MALDI MSI (Figure 4, lower half). The optical image is shown in Figure 4i. With IR-MALDI-DBD, the same signals increased in intensity, enabling visualization of the compound distribution in tissue. In line with the described results for analytical standards and mouse brain measurements using IR-MALDI-DBD, the accurate mass measurements revealed that these ions did not carry sodium or potassium but were [M + H]^+^ or [M – H_2_O + H]^+^ ions. Based on annotated sum formulae, the ions contained 27 (Figure 4a,d), 28 (Figure 4c), 29 (Figure 4e–g), and 30 (Figure 4b) carbon atoms and mainly differed in the overall number of hydrogen and oxygen atoms. Therefore, all of the imaged ions most likely belonged to the steroid compound class. Potassiated PC(34:2) is shown in Figure 4h for comparison, confirming that compounds primarily ionized in IR-MALDI were only marginally affected by activation of the DBD unit. Some of the analytes were distributed across the entire tissue section, covering the gut lumen/plant region as well as the caterpillar tissue (Figure 4a,c,d,h). In contrast, other compounds were mainly localized in the caterpillar tissue (Figure 4f,h) or the gut lumen (Figure 4b,e) regions of the tissue section.

While confident annotation of these signals is only possible when performing on-tissue MS/MS and LC-MS/MS experiments, we checked the assignment of these compounds for consistency. Only compounds that are known to be part of the Kegg pathway and are curated in the LIPID MAPS or Metlin databases were considered. For *m*/*z* 385.3465 shown in Figure 4a, only vitamin D3, previtamin D3, cholest-4-en-3-one, desmosterol, and 7-dehydrocholesterol out of 75 potential annotations were in line with our additional annotation criteria. A distribution of these four compounds in the entire tissue section, as shown in Figure 4a, is also consistent with the physiological relevance for these steroids as they are all essential metabolites for animals and plants. In contrast, Figure 4b shows only signal intensities above the noise level in the plant/gut lumen region of the sample. Compounds known to be present only in plants are lanosterol, cycloartenol, cycloeucalenol, ß-amyrin, and lupeol. The assignment of *m*/*z* 427.3934 is also consistent with the distribution of *m*/*z* 425.3778 shown in Figure 4g. The signal only appears in the plant/gut lumen region of the sample, similar to Figure 4b, and multiple metabolites connected to annotations for *m*/*z* 427.3934 are assigned to Figure 4g. One of these assignments is lupenone, known to be present in many plants together with lupeol. Even though the vast majority of signals cannot be assigned to a single chemical entity based on accurate MSI measurements alone, these results show that nonpolar metabolites are efficiently ionized and imaged in complex biological tissues with the new ion source. In our case study, this allows to discern sterol compounds associated with plant materials within the plant/gut lumen region, and compounds only found in *Danaus plexippus* tissue as showcased in Figure S10 are not detectable with IR-MALDI MSI.

## Conclusions

We described a coaxial atmospheric-pressure IR-MALDI MSI setup modified with a DBD post-ionization source integrated into the mass spectrometric transfer capillary. Based on experiments with authentic standards and well-characterized samples, our results indicate that the DBD extension of the IR-MALDI source significantly increases the number of compound classes imageable in a single MSI run. Polar compounds readily detected with IR-MALDI are not or only marginally influenced in signal intensity by activation of the DBD unit, whereas nonpolar metabolites are enhanced in signal intensity by up to 4 orders of magnitude or even become detectable above the noise level upon DBD activation. This is in line with reports of IR-MALDI and DBD characteristics favoring ionization of polar and nonpolar metabolites, respectively. This suggests that the primary IR-MALDI ionization event is decoupled from the DBD ionization within the transfer capillary and that DBD primarily ionizes IR-laser-desorbed neutrals. The enhancement in analytical performance characteristics of the IR-MALDI-DBD MSI setup compared to IR-MALDI MSI enabled imaging experiments of metabolites in the mouse brain and *D. plexippus* caterpillar sections with lateral resolutions down to 20 μm. To the best of our knowledge, this is the highest reported lateral resolution for IR-laser-based MSI of metabolites. Consistent with studies of standards and well-known samples, the visualized compounds from tissues were assigned to nonpolar compound classes such as sterols, FAs, MGs, and DGs, not accessible with IR-MALDI MSI. This documents the increased number of substance classes imaged with IR-MALDI-DBD under atmospheric-pressure conditions and without sample pretreatment in a single MSI experiment compared to IR-MALDI. We see this as a step toward covering a large set of all metabolites within complex biological samples in a single MSI experiment. Additionally, the modular nature of the DBD device will most likely benefit other MSI modalities that are biased toward ionization of polar compounds.

Future research efforts will be directed toward (a) improving the lateral resolution of the IR-MALDI setup, (b) testing DBD gases or gas mixtures to maximize signal enhancement upon post-ionization, and (c) coupling our DBD setup to other available MSI sources. Our goal is to extend our research efforts in lipid metabolism toward sterols and neutral lipids, using the new hybrid MSI setups. These compound classes are typically challenging to detect with most MSI methodologies but have been linked with parasite infestation^51^ and modulation of immune response.^52^ Consequently, we envision to study the local response of sterol and neutral lipid levels in the context of neglected tropical diseases (NTDs) with aid of state-of-the-art chromatographic and here-presented DBD-based MSI methodologies in the near future.

## Supporting Information Available

The Supporting Information file contains detailed information about technical setups, optimization parameters, and measurements, as well as some example spectra and images (PDF)

## Notes

Animal parts used for this study were obtained by veterinary experts under ethical approval by Justus Liebig University (Approval code: 571_M, Approval date: 21 February 2017).
