# Supplementary material for: IR-MALDI Mass Spectrometry Imaging with Plasma Post-Ionization of Nonpolar Metabolites
Source: Anal Chem. 2022 Nov 10;94(46):16086–94. doi: 10.1021/acs.analchem.2c03247 (PMC9685590; doi:10.1021/acs.analchem.2c03247)
Supplement: Supplementary file 1 — ac2c03247_si_001.pdf [file ac2c03247_si_001.pdf]

Supporting Information

IR-MALDI mass spectrometry imaging with plasma post-ionization of non-polar metabolites

Julian Schneemann,[a] Karl-Christian Schäfer,[b] Bernhard Spengler,[a]* Sven Heiles[a,c,d]*

[a] *Institute of Inorganic and Analytical Chemistry, Justus Liebig University Giessen, 35392 Giessen, Germany*
[b] *TransMIT GmbH, 35392 Giessen, Germany*
[c] *Leibniz-Institut für Analytische Wissenschaften - ISAS - e.V., Otto-Hahn-Straße 6b, 44139 Dortmund, Germany*
[d] *Lipidomics, Faculty of Chemistry, University of Duisburg-Essen, Universitätsstrasse 5, 45141 Essen, Germany*

Table of contents

1. Figure S1: Deoxycholic acid on glass slide after IR-MALDI experiments and detailed description of the ion source S2
2. Figure S2: Burn pattern (ablation crater) after IR-MALDI experiments on sprayed deoxycholic acid on glass slide. S4
3. Figure S3: Parameter optimization of the DBD post-ionization source. S5
4. Figure S4: MALDI-MS^2 spectrum of [capsaicin + $H]^+$ and [dihydrocapsaicin+ $H]^+$ S6
5. Figure S5: MALDI-MS^2 spectrum of vitamin D_3 recorded in laser + LTP mode S7
6. Figure S6: Sum spectrum of frozen mouse brain and of frozen mouse liver tissue, spiked with ergosterol in laser+LTP mode and laser-only mode S8
7. Figure S7: High-resolution MSI measurement of mouse brain with a step size of 20 µm. S9
8. Figure S8: Putative structures of the imaged compounds from Figure 3. S10
9. Figure S9: Optical image of a section of monarch butterfly (*Danaus plexippus*) caterpillar, fed on tweedia (*Oxypetalum coeruleum)* after MSI measurement, showing ablation spots S11
10. Figure S10: RGB image of monarch butterfly (D. plexippus) caterpillar section, fed on tweedia (O. coeruleum) and optical image. S12

*Address correspondence to

Prof. Dr. Bernhard Spengler:
Institute of Inorganic and Analytical Chemistry, Justus Liebig University Giessen, Heinrich-Buff-Ring 17, 35392 Giessen, Germany, e-mail: bernhard.spengler@anorg.chemie.uni-giessen.de

or to

Prof. Dr. Sven Heiles:
ISAS e.V., Otto-Hahn-Straße 6b, 44227 Dortmund, Germany, e-mail: sven.heiles@isas.de

Details of the IR-MALDI source with an in-capillary DBD unit

In the ion source, laser light with a wavelength of 2.94 μm generated by an optical parametric oscillator (GWU Lasertechnik GmbH, Erftstadt, Germany) pumped by a Nd:YAG laser system (SpitLight 400, InnoLas Laser GmbH, Krailling, Germany), which constantly triggers the flashlamps at 20 Hz and synchronizes the laser with the mass spectrometer via the Q-switch opening, was used for all experiments. Mirrors, attenuators, and lenses guided the laser light into a centrally bored custom-made objective lens that is engulfing the inlet capillary of the mass spectrometer. The laser energy per pulse available for sample desorption and ionization ranged from 2 to 23 μJ/pulse and was measured with a laser power meter (FieldMaxII-TOP, Coherent, Dieburg, Germany). By adjusting the laser energy settings, effective laser ablation spots with 25-30 μm diameter were routinely obtained (Figure S1-2). Prior to experiments, the sample holder, equipped with a Peltier system (TES1-127025, TRU Components) and a Pt100 resistance thermometer coupled to a solid-state relais (XSSR-DA2420, Zhejiang Xurui Electronics Co.,LTD), was cooled to 14 to 15 °C to condense air humidity onto the sample for enabling IR-MALDI. All experiments were performed with ten laser pulses per sampling spot. The MS inlet capillary was arranged in the center of the focusing objective because a geometry normal to the sample surface and coaxial between laser incidence and ion transmission direction is known to maximize the capture of desorbed neutrals and ions from the sampled surface.[1] The MS inlet capillary was equipped with a T-piece, which connected the setup to the DBD ion source, consisting of a glass casing with an outer diameter of 3 mm, an inner diameter of 1.6 mm, a grounded inner tungsten wire electrode and an outer copper foil (**Figure 1a**, 5-7). The copper foil was 0.9 cm wide and had a distance of 1.0 cm to the end of the glass capillary. The glass capillary of the DBD source overlaped about 2 mm with the metal part of the T-piece, which had an outer diameter of 1.59 mm and a length of 3.0 cm. The T-piece was positioned with a distance of 1.4 cm from the objective lens and 1.9 cm from the end of the transfer capillary. The DBD plasma was operated with He gas and reactive species were transported into the MS inlet capillary to interact with desorbed/ionized molecules. Importantly, the desorbed analytes do not directly interact with the DBD plasma but with the reactive species generated in the plasma. Molecules ionized during IR-MALDI or DBD were transported into an orbital trapping mass analyzer and were simultaneously detected.

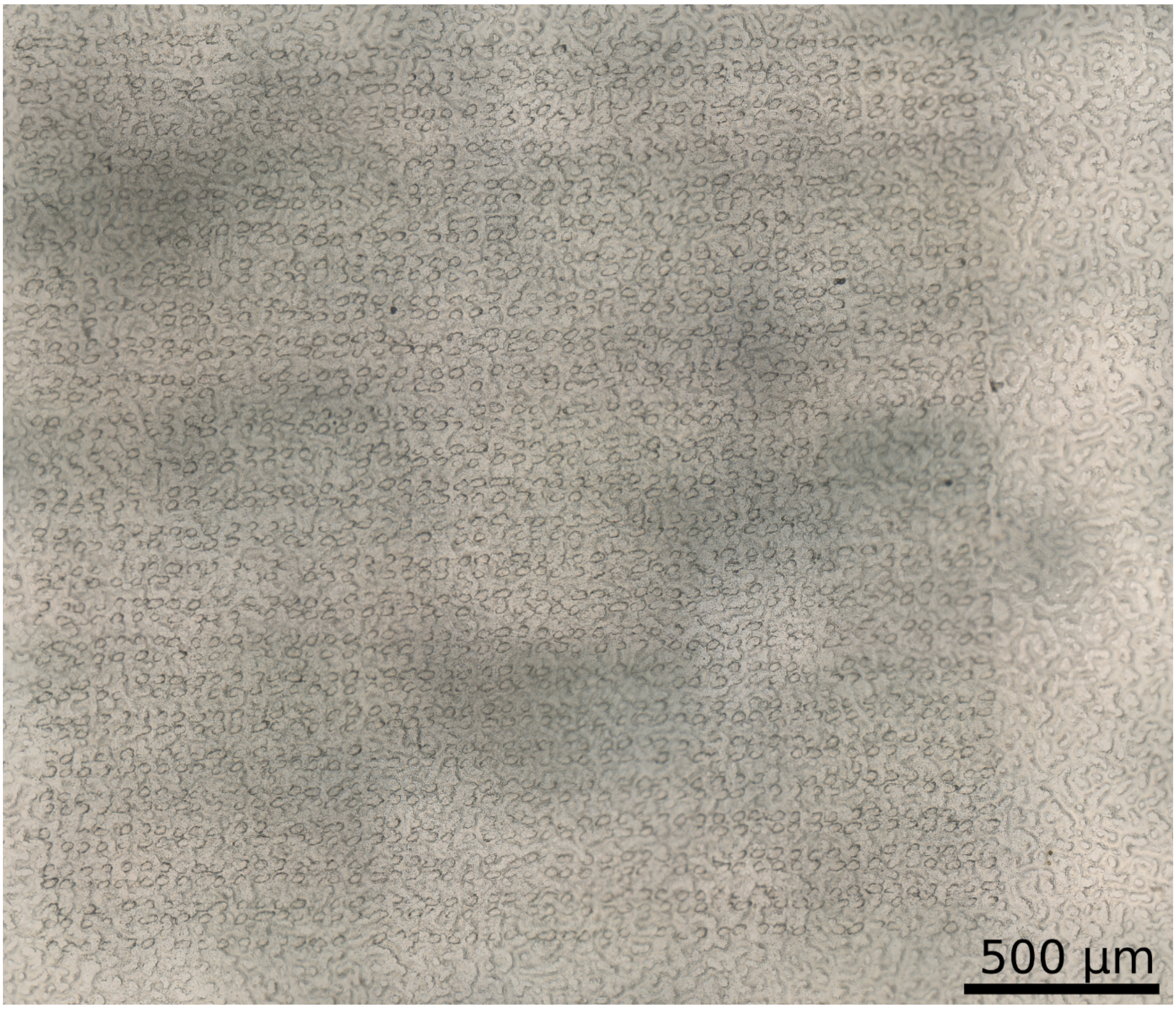

Figure S1: Optical image of sprayed deoxycholic acid on glass slide with ablation spots after IR-MALDI experiments.

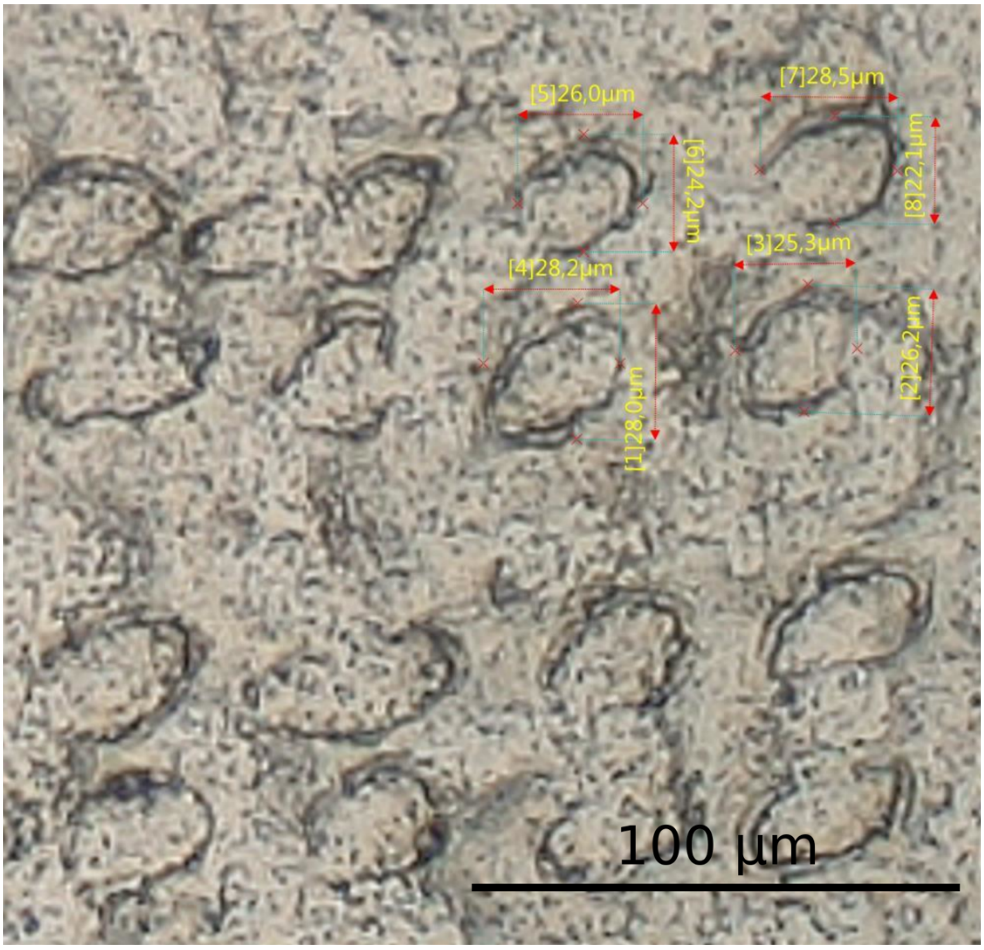

Figure S2: Burn pattern (ablation crater) after IR-MALDI experiments on sprayed deoxycholic acid on glass slide.

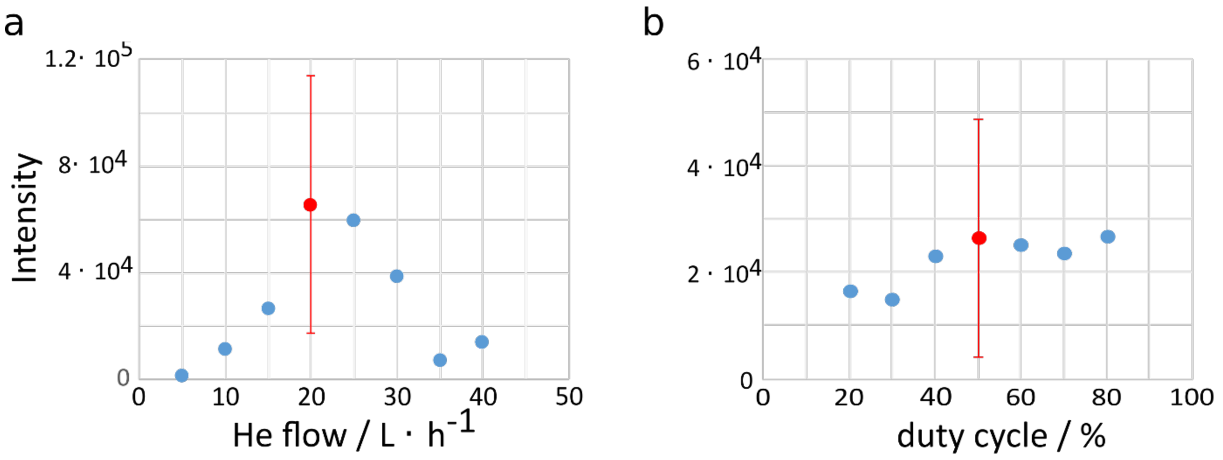

Figure S3: Parameter optimization with homogenously sprayed deoxycholic acid [M – 2 H_2O + H]+ for varying (a) the helium flow and (b) the duty cycle of the DBD post-ionization source.

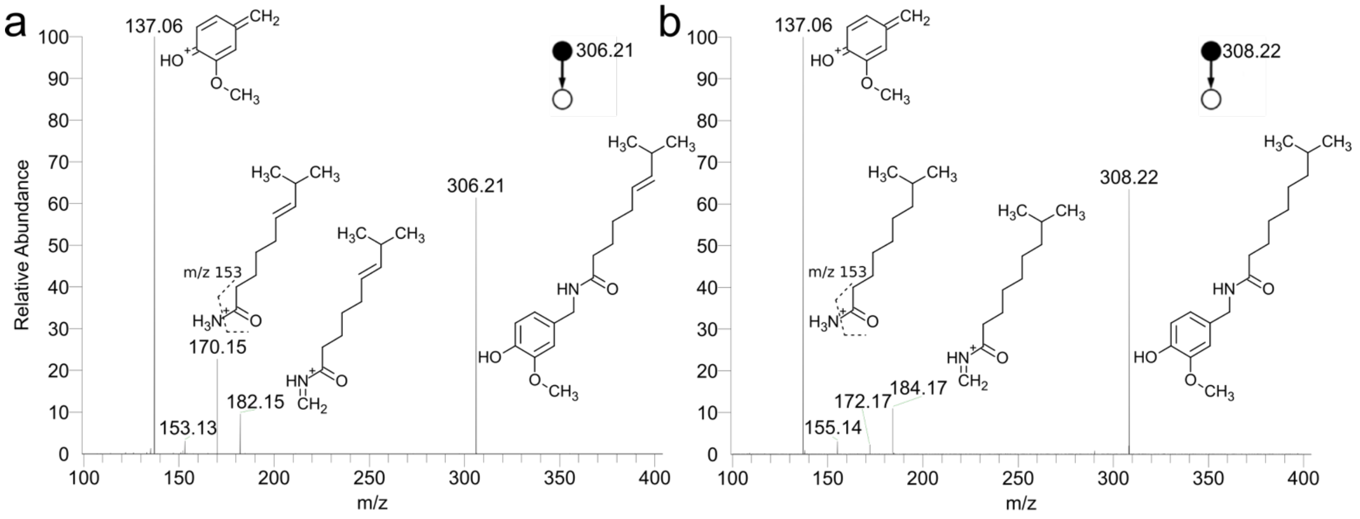

Figure S4: MALDI-MS^2 spectrum of $[capsaicin + H]^+$ (a) and $[dihydrocapsaicin + H]^+$ (b) from chili pepper "*Carolina Reaper*", recorded in laser+LTP mode.

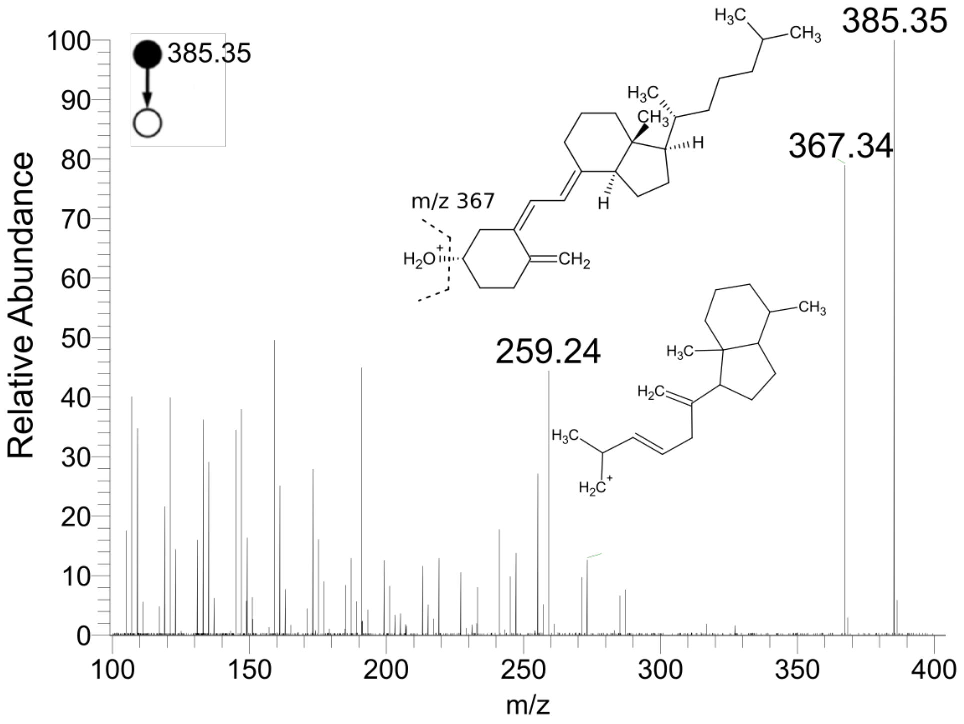

Figure S5: MALDI-MS^2 spectrum of vitamin D_3 recorded in laser+LTP mode.

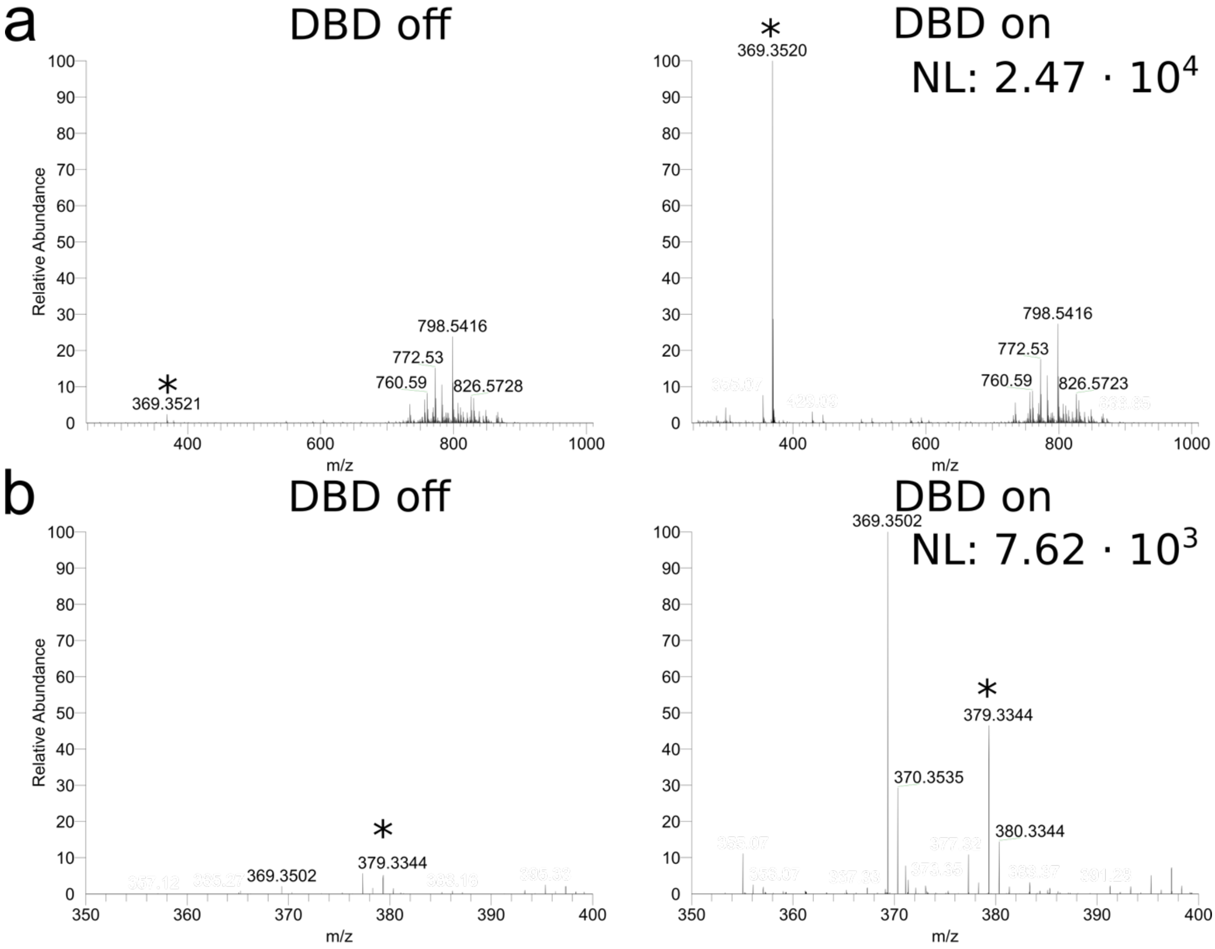

Figure S6: a: Sum spectra of frozen mouse brain in laser+DBD mode on the left and laser-only mode on the right. [Cholesterol – H_2O + H]$^+$ (*m/z* 369.3516) is indicated with an asterisk. b: Sum spectra of frozen mouse liver spiked with ergosterol in laser+LTP mode on the left and laser-only mode on the right. [Ergosterol – H_2O + H]$^+$ (m/z 379.3359) is indicated with an asterisk.

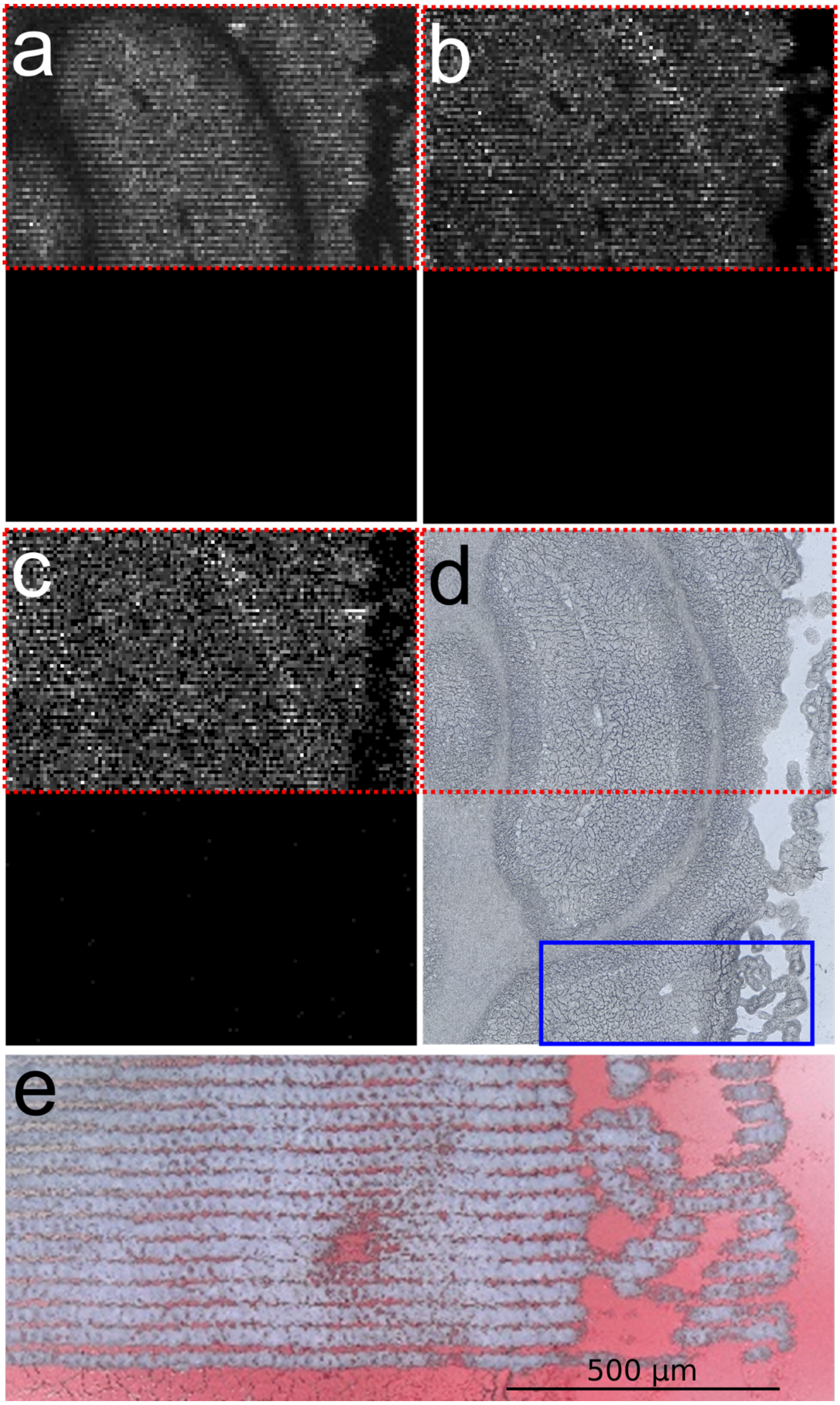

Figure S7: High-resolution MSI measurement of mouse brain with a step size of 20 µm. a: MS image of [stearic acid + H]$^+$ (*m*/*z* 285.2789), b: MS image of [cholesterol – H_2O + H]$^+$ (*m*/*z* 369.3516), c: MS image of [cholecalciferol + H]$^+$ (*m*/*z* 385.3465), d: optical image before the measurement and e: optical image of the ablation spots of the blue rectangle from d after the measurement. The red dotted rectangles indicate laser+LTP mode.

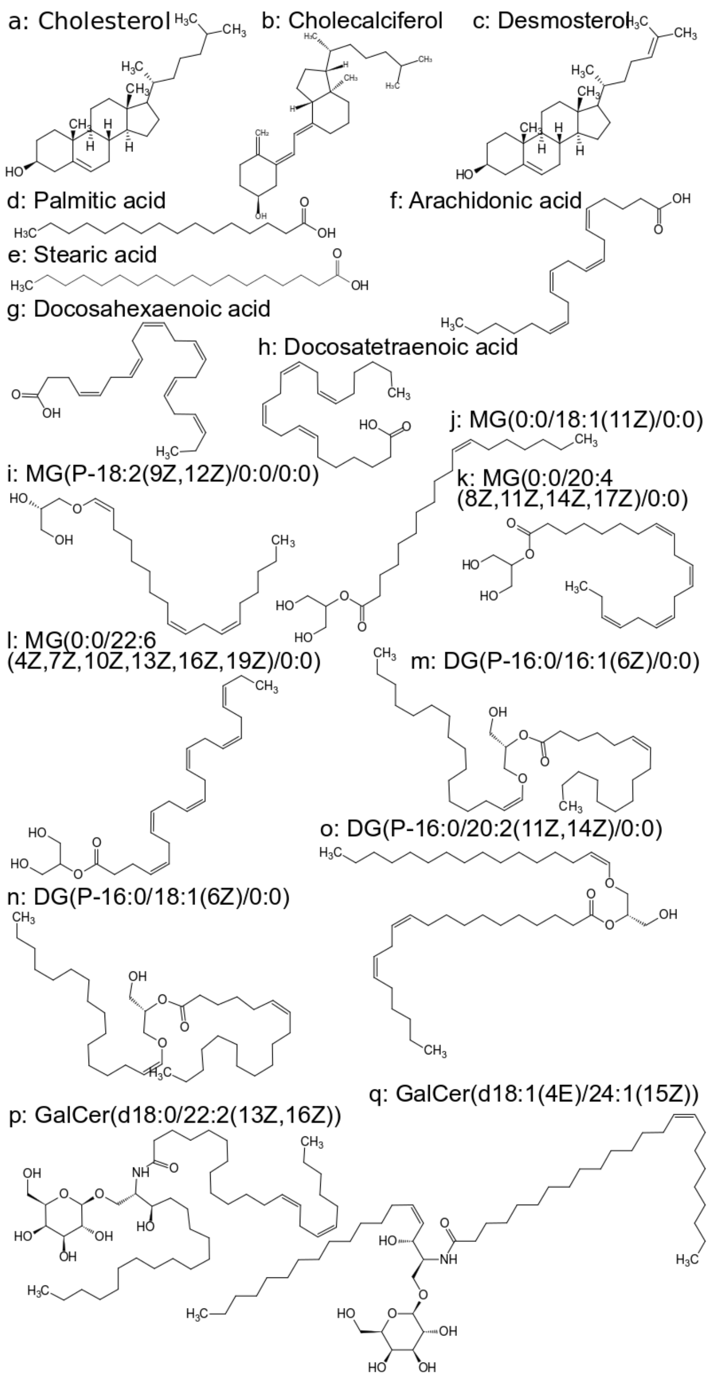

Figure S8: Putative structures of the imaged compounds from Figure 3. The assignments are based on accurate mass measurements.

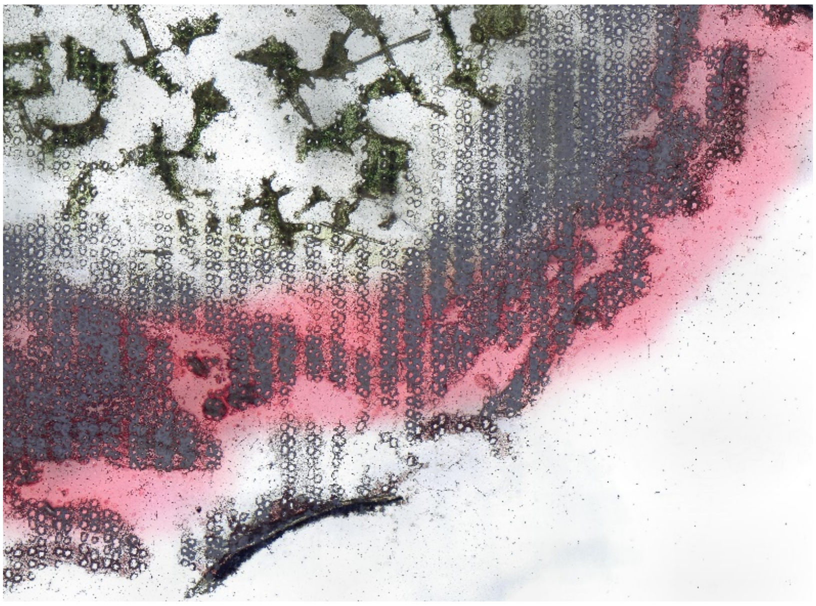

Figure S9: Optical image of a section of monarch butterfly (*Danaus plexippus*) caterpillar, fed on tweedia (*Oxypetalum coeruleum)* after the MSI measurement, showing ablation spots.

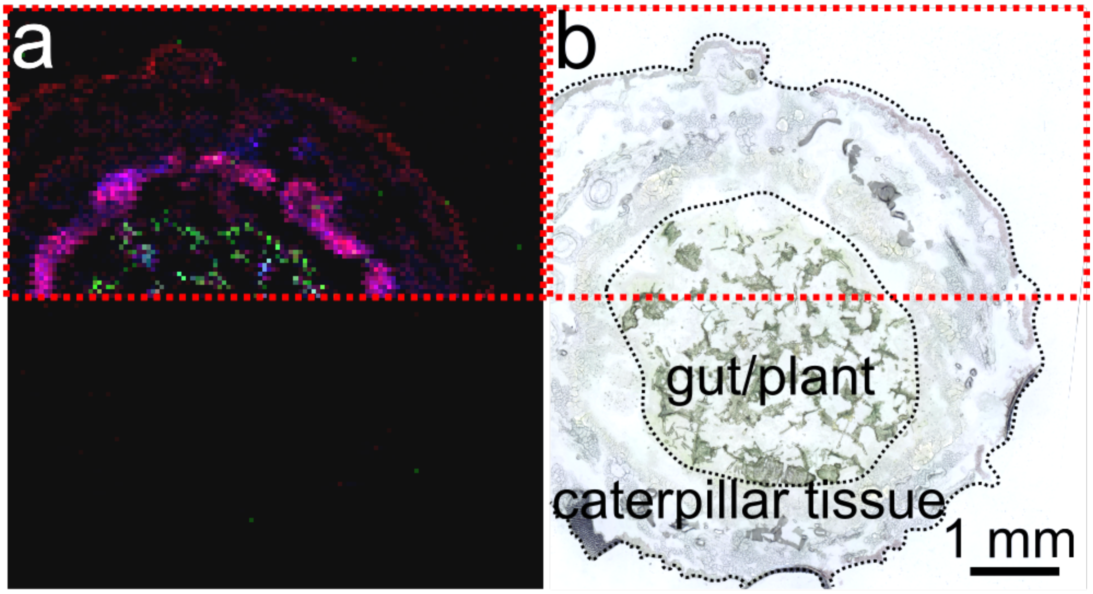

Figure S10. a: RGB image of monarch butterfly (*D. plexippus*) caterpillar section, fed on tweedia (*O. coeruleum*). The distributions of $[C_{29}H_{49}O]^+$ (*m/z* 413.3778) (red), $[C_{31}H_{53}O]^+$ (*m/z* 441.4091) (green), $[C_{18}H_{31}O_2]^+$ (*m/z* 279.2319) (blue) are overlayed. b: Optical image of the tissue section before MSI experiments. The red dotted rectangles indicate the part of the tissue scanned with IR-MALDI-DBD MSI.

References

(1) Spengler, B.; Bökelmann, V. Angular and time resolved intensity distributions of laser-desorbed matrix ions. *Nucl. Instrum. Methods Phys. Res. B* **1993**, *82*, 379–385.
